# Biomechanical Assessment Tools for Injury Risk Prediction and Return-to-Sport Evaluation in Athletes: A Systematic Review

**DOI:** 10.7759/cureus.93210

**Published:** 2025-09-25

**Authors:** Adel A Alahaidib, Hind Y Alyousef, Murad A Sharif, Abdulaziz K Alsulaiman, Thamer S Alharthi, Hatim I Aljohani, Muteb K Almutairi, Nawwaf S Alghamdi, Khaled G Almutairi, Abdullah O Alammari, Abdulmohsin A Almehizia

**Affiliations:** 1 Orthopedic Surgery, Dallah Hospital, Riyadh, SAU; 2 College of Medicine, Al Jouf University, Al Jouf, SAU; 3 College of Medicine, Jazan University, Jazan, SAU; 4 College of Medicine, King Abdulaziz University, Jeddah, SAU; 5 College of Medicine, Bisha University, Bisha, SAU; 6 College of Medicine, King Saud bin Abdulaziz University for Health Sciences, Jeddah, SAU; 7 College of Medicine, Shaqra University, Riyadh, SAU; 8 College of Medicine, Qassim University, Qassim, SAU; 9 Orthopedic Surgery, King Fahad Medical City, Riyadh, SAU

**Keywords:** acl injury, biomechanics, force plate, injury risk, kinematics, limb symmetry, motion analysis, rehabilitation, return-to-sport, screening tools

## Abstract

Anterior cruciate ligament (ACL) injuries, particularly non-contact types, remain a major concern for athletes. Biomechanical screening tools such as 3D motion capture and force plates have advanced risk assessment and return-to-sport evaluation, yet their predictive validity and clinical applicability across diverse athletic populations remain unclear. This systematic review, conducted in accordance with PRISMA 2020 guidelines, searched four databases (PubMed, Cochrane Library, Scopus, Web of Science) up to July 25, 2025, and included original studies assessing athletes with biomechanical tools for injury risk or return-to-sport readiness. Of 4,787 records identified, eight studies met the criteria. Most utilized motion analysis or force plates to evaluate landing kinematics, limb symmetry, and postural control. Key findings showed that reduced quadriceps strength symmetry, psychological unreadiness, and compensatory kinematic deviations were strongly associated with delayed or unsafe return-to-sport. While sagittal-plane metrics demonstrated high reliability, coronal and transverse measures were less consistent, and proprietary force-plate scores, such as Sparta Science metrics, showed limited predictive value unless used within multifactorial frameworks. Overall, biomechanical assessments provide meaningful insights into injury risk and rehabilitation progress, particularly when emphasizing strength symmetry and kinematic consistency, but reliance on single metrics or composite scores without contextual interpretation may restrict clinical utility. Future research should focus on longitudinal designs and standardized protocols to enhance predictive accuracy.

## Introduction and background

Non-contact anterior cruciate ligament (ACL) injuries often occur during dynamic sports movements such as jump-landing and rapid deceleration, particularly in athletes engaged in high-demand activities [1,2]. The risk is especially elevated in female athletes due to distinct neuromuscular and anatomical predispositions [2,3]. Motion capture systems and force plate technologies have become standard tools to quantify biomechanical variables, including joint angles, ground reaction forces, and impact loading patterns, enabling early identification of movement deviations linked to ACL injury risk [4].

Prospective and scoping reviews indicate that specific biomechanical markers-particularly those from drop vertical jump tests-can predict ACL injury, with seven of 11 prospective studies reporting significant associations [4]. Common predictors include increased knee valgus, reduced hip control, and asymmetric force application. Reliability studies across multiple motion-analysis centers demonstrate strong consistency in sagittal-plane measures, though coronal and transverse data remain more variable, underscoring the need for standardized protocols [5].

Recent evidence suggests that combining biomechanical screening with neuromuscular and psychological profiling enhances predictive accuracy. For example, latent profile analysis shows that intervention effects on knee abduction moment differ across individual risk profiles [2,6]. Increasingly, experts advocate for in-field biomechanical tools capable of capturing key kinetic and kinematic data outside laboratory settings. With the maturation of consumer-grade and machine learning-based systems, their potential to improve real-world injury surveillance and prevention strategies is becoming more evident.

## Review

Methods

Literature Search Strategy

This systematic review followed Preferred Reporting Items for Systematic Reviews and Meta-Analyses (PRISMA) 2020 guidelines [7]. We searched PubMed (n=884), Cochrane Library (n=362), Scopus (n=1,896), and Web of Science (n=1,645) from inception to July 25, 2025. The search combined controlled vocabulary and free-text terms for biomechanical assessment (e.g., biomechanical*, kinematic*, kinetic*, “motion analysis”, “force plate”, “3D motion capture”, wearable), athletes (e.g., athlete*, sport*, footballer*, runner*, soccer, basketball) and injury (e.g., injur*, “injury risk”, “return to sport”). Strategies were adapted to each database and limited to English-language human studies.

Eligibility Criteria

Selection used the PICOS framework (Population, Intervention, Comparison, Outcome, Study design) [8]. We included original English-language studies of athletes (any sport/level/age) that used biomechanical tools (motion analysis, force plates, wearables) to assess injury risk or return-to-sport, reported kinematic/kinetic or injury-related predictive outcomes, and included relevant comparisons (contralateral limb, healthy controls, or return-to-sport outcomes). Excluded were non-athlete studies, papers not focused on injury prediction, conference abstracts without full texts, non-English articles, and review/commentary pieces.

Study Selection and Data Extraction

Two reviewers independently screened titles/abstracts and full texts; disagreements were resolved through discussion with a third reviewer. From the included studies, we extracted study ID, country, design, setting, sample size and demographics, biomechanical tool, measured variables, and key findings. Extraction was performed independently by two reviewers with arbitration by a third when required.

Quality Appraisal

Two reviewers independently assessed methodological quality using the Newcastle-Ottawa Scale for cohort and observational studies [9], scoring selection, comparability, and outcome assessment (maximum 9 stars): high (7-9), moderate (5-6), or low (≤4). Any disagreements were resolved by consensus.

Results

Study Selection

**Figure 1:**
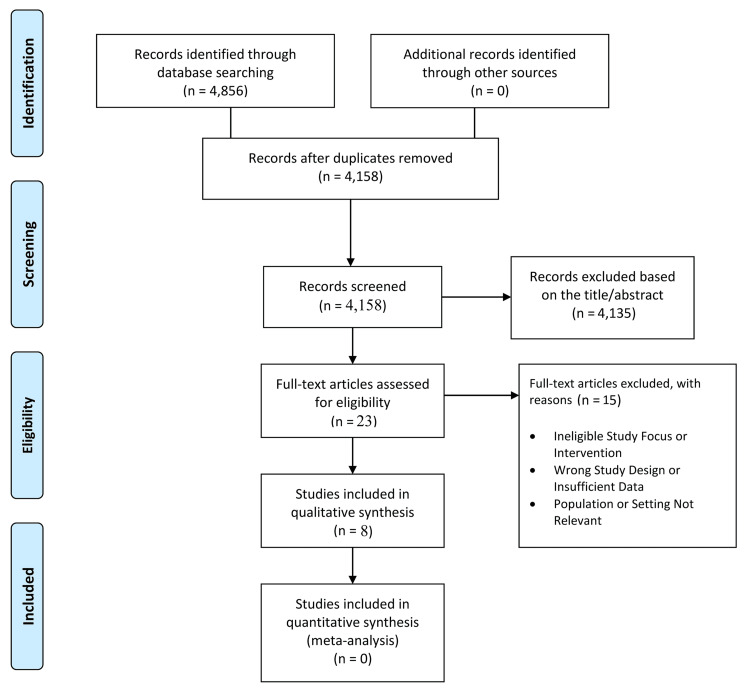
Preferred Reporting Items for Systematic Reviews and Meta-Analyses (PRISMA) flow diagram of the study selection process

The study selection process adhered to PRISMA 2020 guidelines (Figure 1). An initial total of 4,856 records were identified through comprehensive database searches. After removing duplicates, 4,158 unique records remained for title and abstract screening. Of these, 4,135 records were excluded for reasons such as irrelevance to biomechanical screening, non-athlete populations, or lack of prognostic intent. This left 23 full-text articles for detailed eligibility assessment. Following full-text review, 15 studies were excluded due to various reasons. Ultimately, eight studies met the inclusion criteria and were included in the qualitative synthesis, with no studies qualifying for quantitative synthesis due to heterogeneity in methods and outcomes.

Study Characteristics

The characteristics of the included studies are summarized in Table 1.

**Table 1:** Summary of biomechanical studies investigating anterior cruciate ligament reconstruction and injury risk Abbreviations: ACLR = anterior cruciate ligament reconstruction, RTS = return to sport, OA = osteoarthritis, LSI = limb symmetry index, DL-CMJ = double-leg countermovement jump, SL-CMJ = single-leg countermovement jump, SL-RH = single-leg repeated hop, DVJ = drop vertical jump, ROM = range of motion, ML/AP = mediolateral/anteroposterior, TSLH = timed single-leg hop, SEBT = star excursion balance test, UPDB = unipedal balance test, MSKI = musculoskeletal injury, RFD = rate of force development, MSK Health Score = musculoskeletal health score (Sparta Science system).

Study ID	Country	Study Design	Setting	Sample Size & Demographics	Biomechanical Tool Used	Kinematic Variables Measured	Study Results & Key Findings
Hewett et al. [5]	USA	Multicenter reliability study	Three motion labs (Cincinnati, OSU, Kentucky)	25 female volleyball players, mean age 15.3 ± 1.0 yrs	3D motion capture & force plates	Peak hip, knee, and ankle angles/moments (sagittal, coronal, transverse planes) during DVJ	Demonstrated excellent within-site reliability; sagittal plane variables were most reliable, supporting multicenter ACL risk screening.
Zhou et al. [10]	China	Cross-sectional observational	Rehabilitation research facility	45 male ACLR patients, mean age 28 ± 0.8 yrs, 8.3 ± 1.5 months post-op	Opti-Knee 3D motion analysis system	ROM, maximum angle, angle at initial contact (sagittal, frontal, transverse planes)	Psychological readiness and quadriceps symmetry were associated with symmetrical knee kinematics, which are key for ACL injury prevention and reducing OA risk.
Hart & Chumanov [11]	USA	Retrospective cohort study	Rehabilitation centers (Wisconsin)	49 male basketball players (14–25 yrs), post-ACLR	Dual force plate (AMTI, 1000 Hz)	Jump height, impulse, flight time/contact time, peak forces	Quadriceps LSI <90% was linked to asymmetry in DL-CMJ and SL-RH, but not in SL-CMJ. Authors recommend multi-test RTP screening.
Baltich et al. [12]	Canada	Cross-sectional comparative	Sports medicine centre (Calgary)	100 youth/young adults (50 with prior knee injury, 50 matched controls), age 15–26 yrs	Kistler force plate, TSLH, SEBT, UPDB	ML/AP excursion, ellipse area, path length, hop distance, reach distance, sway time	Previously injured group showed greater sway and lower TSLH; force plate balance was strongly associated with injury history.
Norouzi et al. [13]	Iran	Cross-sectional comparative	Physiotherapy clinics & football clubs	42 male footballers (27 ACLR, 15 healthy); RTS vs. fail RTS groups	3D motion capture (Qualisys, 240 Hz), Visual3D	Hip, knee, and ankle angles in three planes during landing	RTS group demonstrated reduced hip abduction; fail RTS group showed reduced ankle inversion. RTS criteria may miss key biomechanical risk factors.
Hando et al. [14]	USA	Retrospective cohort study	Air Force training center	823 male trainees, mean age 22.7 ± 3.9 yrs	Sparta Science force plate system	Load, Explode, Drive, Sparta Score, MSK Health Score	Sparta metrics showed excellent reliability (ICC > 0.9) but poor predictive value for MSKI; age and baseline fitness were stronger predictors.
Pontillo et al. [15]	USA	Retrospective cohort study	Division I collegiate athletic program	16 ACL injuries (8 male, 8 female), 262 controls; athletes from seven sports	Sparta Science force plate system	Load (eccentric RFD), Explode (concentric force), Drive (concentric impulse), and ratios	Explode and Drive metrics predicted ACL injury (p = 0.03); injured athletes showed altered jump kinetics.
O’Malley et al. [16]	Ireland & UK	Cross-sectional	Orthopaedic hospital	118 ACLR patients, 44 controls; male field-sport athletes aged 24–26 yrs	3D motion analysis, force plates, isokinetic dynamometer	SL-CMJ height, peak power, hip/knee/ankle joint contribution, knee torque, LSI	ACLR patients had reduced SL-CMJ height and torque; hip compensation was observed. RTS benchmarks: >17 cm SL-CMJ, >260% torque, >90% LSI for strength and jump.

Zhou et al. [10] employed the Opti-Knee 3D motion analysis system to evaluate 45 male patients post-ACL reconstruction. Their cross-sectional observational study demonstrated that symmetrical knee kinematics were associated with both psychological readiness and quadriceps strength symmetry. The integration of psychological and biomechanical factors highlighted the multifactorial nature of injury recovery and prevention.

In a retrospective cohort study, Hart and Chumanov [11] assessed 49 male basketball players post-anterior cruciate ligament reconstruction (ACLR) using high-frequency AMTI force plates. By analyzing countermovement and repeated hop tasks, they found that athletes with a quadriceps limb symmetry index (LSI) below 90% exhibited significant force asymmetries, especially in dynamic tasks. Their results underscored the inadequacy of using a single performance test for return-to-play clearance and advocated for multidimensional screening protocols.

A large-scale multicenter reliability investigation was conducted by Hewett et al. [5] using synchronized 3D motion capture and force plate technology across three U.S. institutions. Their evaluation of drop vertical jump kinematics in adolescent female volleyball players revealed excellent within-site reliability, particularly for sagittal-plane measures. This validated the feasibility and consistency of biomechanical injury-risk screening across institutions.

Baltich et al. [12] conducted a cross-sectional comparative study on 100 youth and young adults, half of whom had a prior knee injury. Using a combination of Kistler force plates and field-based tests like the single-leg hop and balance assessments, they identified significantly greater postural sway and reduced functional performance in the injured group. These findings supported the role of proprioceptive and postural stability metrics in profiling injury risk. In a similar vein, Norouzi et al. [13] examined biomechanical landing profiles in 42 Iranian male footballers using a 3D Qualisys motion capture system. The study revealed reduced hip abduction in RTS-cleared athletes and diminished ankle inversion in those who failed RTS. The findings raised concerns about the sufficiency of conventional RTS criteria, emphasizing the potential for biomechanical variables to reveal lingering risk factors undetected by standard clinical assessments.

The Sparta Science force plate system was featured in two U.S.-based retrospective studies. Hando et al. [14] analyzed jump kinetics in over 800 military trainees, reporting high test-retest reliability for Sparta-derived variables (Load, Explode, Drive), yet limited predictive validity for musculoskeletal injuries. This highlighted the limitations of relying solely on composite scores and suggested that contextual factors such as age and prior injury history may better inform risk profiles. Complementing this, Pontillo et al. [15] evaluated jump data from over 270 NCAA athletes and found that altered Explode and Drive values significantly predicted ACL injury. Despite the retrospective design and lack of exposure control, it added to the evidence suggesting that subtle force production imbalances may precede injury events.

Finally, O’Malley et al. [16] compared kinetic and isokinetic data between 118 ACLR athletes and 44 controls. Using motion capture, force plates, and isokinetic dynamometry, they identified decreased single-leg jump height, lower torque, and compensatory hip mechanics in the ACLR group. The authors proposed quantitative return-to-play thresholds (e.g., >260% torque, >17 cm jump height, >90% LSI) to guide safer decision-making.

Quality Assessment

The quality assessment revealed that most studies demonstrated strong methodological rigor in the selection domain, with all eight studies-Zhou et al. [10], Hart and Chumanov [11], Hewett et al. [5], Baltich et al. [12], Norouzi et al. [13], Hando et al. [14], O’Malley et al. [16], and Pontillo et al. [15]-receiving full stars for representativeness of the exposed cohort, selection of the non-exposed cohort, and ascertainment of exposure. Most studies, except for Hart and Chumanov [11] and Baltich et al. [12], ensured that the outcome of interest was not present at baseline. In terms of comparability, Zhou et al. [10], Hewett et al. [5], Norouzi et al. [13], and Hando et al. [14] controlled for confounding using one or more key variables, while Hewett et al. [5] and Zhou et al. [10] also addressed additional confounders, earning full stars in this domain. Outcome assessment was generally adequate, with all studies using objective biomechanical or clinical criteria, but longitudinal strength varied. Only Hewett et al. [5] reported long-term follow-up with adequate retention, earning a full score of 9/9. Zhou et al. [10], Norouzi et al. [13], and Hando et al. [14] scored 7/9, reflecting minor gaps in follow-up duration or completeness. O’Malley et al. [16] and Pontillo et al. [15] scored 6/9, largely due to limited follow-up data, while Hart and Chumanov [11] and Baltich et al. [12] scored lowest (5/9), primarily due to absent follow-up periods and limited control for confounders. Overall, the majority of studies exhibited moderate to high methodological quality, with Hewett et al. [5] standing out as the most robustly designed (Table 2).

**Table 2:** Newcastle–Ottawa Scale quality assessment of included cohort studies The NOS evaluates nonrandomized studies in three domains: Selection (representativeness of exposed cohort, selection of non-exposed cohort, ascertainment of exposure, outcome not present at baseline), Comparability (control for confounding), and Outcome (outcome assessment, adequacy of follow-up). Scores range from 0 to 9, with higher scores indicating better methodological quality. “Yes” indicates that the criterion was fulfilled; “No” indicates that it was not.

Study (Author [Ref No.])	Representativeness of Exposed Cohort	Selection of Non-Exposed Cohort	Ascertainment of Exposure	Outcome Not Present at Start	Comparability of Cohorts	Additional Confounders Controlled	Outcome Assessment	Long Enough Follow-Up	Adequacy of Follow-Up	Total /9
Hewett et al. [5]	Yes	Yes	Yes	Yes	Yes	Yes	Yes	Yes	Yes	9
Zhou et al. [10]	Yes	Yes	Yes	Yes	Yes	Yes	Yes	No	No	7
Hart & Chumanov [11]	Yes	Yes	Yes	No	Yes	No	Yes	No	No	5
Baltich et al. [12]	Yes	Yes	Yes	No	Yes	No	Yes	No	No	5
Norouzi et al. [13]	Yes	Yes	Yes	Yes	Yes	No	Yes	No	Yes	7
Hando et al. [14]	Yes	Yes	Yes	Yes	Yes	Yes	Yes	No	No	7
Pontillo et al. [15]	Yes	Yes	Yes	Yes	Yes	No	Yes	No	No	6
O’Malley et al. [16]	Yes	Yes	Yes	No	Yes	No	Yes	Yes	No	6

Limb Symmetry, Muscle Strength, and Psychological Readiness

A recurring theme among several studies was the impact of quadriceps strength asymmetry and psychological readiness on injury risk and movement quality. Zhou et al. [10] found that inter-limb differences in quadriceps and hamstring strength were significantly associated with kinematic discrepancies during gait. More importantly, both strength symmetry and psychological readiness (measured via the ACL-RSI score) were linked to improved sagittal plane knee motion, suggesting these factors are essential for restoring symmetrical biomechanics and minimizing the risk of re-injury. Similarly, Hart and Chumanov [11] emphasized that athletes with quadriceps LSI <90% showed poorer force plate metrics across several jump-related variables. These included concentric impulse, eccentric rate of force development, and jump height-highlighting strength deficits as a key biomechanical determinant of inadequate return-to-play readiness. O’Malley et al. [16] reinforced these findings by showing that ACLR athletes demonstrated significantly lower single-leg CMJ height, peak power, and isokinetic torque compared to healthy controls, with clear asymmetries between limbs. Together, these studies suggest that restoring limb symmetry and targeting strength recovery are critical for a safe return to sport.

Biomechanical Asymmetries in Kinematic Profiles

Kinematic deviations during landing and dynamic tasks were consistently observed in at-risk or previously injured athletes. Norouzi et al. [13] reported that ACLR football players-especially those who failed RTS criteria-had reduced hip abduction and ankle inversion angles during landing tasks, despite demonstrating no major differences in knee kinematics. This supports the notion that compensatory patterns may manifest at proximal and distal joints, potentially increasing injury susceptibility even when clinical metrics appear normalized. In the multicenter study by Hewett et al. [5], sagittal-plane peak kinematic and kinetic values showed excellent reliability across three testing sites, whereas coronal and transverse-plane measurements were more variable. These findings affirm the diagnostic consistency of sagittal plane metrics in large-scale screenings and highlight the need for cautious interpretation of off-plane variables due to measurement variability.

Proprietary Force Plate Metrics and Predictive Limitations

The use of proprietary force plate systems like Sparta Science was explored in both Hando et al. [14] and Pontillo et al. [15], though with differing results. Hando et al. found that despite excellent test-retest reliability (ICC > 0.96 for Sparta Load, Explode, and Drive), Sparta MSK Health scores lacked predictive utility for musculoskeletal injury (AUC ~0.52). In contrast, Pontillo et al. [15] demonstrated that altered Explode and Drive metrics could predict ACL injury with small to medium effect sizes (Cohen’s d 0.52-0.61), though the model lacked control for exposure-related confounders. These contrasting findings suggest that while force plate metrics may reveal kinetic imbalances, their predictive value depends on context, covariate control, and implementation in multi-modal screening frameworks.

Balance, Postural Control, and Injury History

Baltich et al. [12] uniquely explored postural stability and field-based balance measures among previously injured and uninjured youth. Injured individuals displayed increased mediolateral sway, larger center of pressure ellipse areas, and poorer single-leg hop performance. The study also showed an association between TSLH distance and balance control, suggesting functional hopping performance could serve as an indirect marker of postural instability linked to prior injury. These results point to the utility of static and dynamic balance tests in identifying neuromuscular deficits not always captured through traditional strength or kinematic assessments.

Discussion

This review highlights the critical role of biomechanical factors in assessing injury risk and guiding rehabilitation in athletes. Consistently, studies indicate that inter-limb asymmetries, muscle strength imbalances, and deviations in movement patterns are associated with delayed return to sport and poorer functional outcomes. Both objective strength measures and psychological readiness emerge as key components in the rehabilitation process, with evidence suggesting that athletes who achieve symmetry in muscle function and demonstrate confidence or readiness are more likely to restore normal movement patterns. These findings underscore the importance of integrating physical and psychological assessments when designing individualized training, injury-prevention, and return-to-play programs, as relying solely on isolated metrics may fail to capture the full risk profile.

Additionally, the evidence points to the value of evaluating compensatory mechanics at proximal and distal joints, as deficits in hip or ankle control can contribute to abnormal force transmission and elevate injury risk. Sagittal-plane kinematic measures appear reliable and practical for large-scale screening, while lateral and transverse-plane data may require careful interpretation due to variability. Similarly, force-plate metrics and balance assessments can provide meaningful insight, especially when embedded within multifactorial models that consider prior injury, strength deficits, and neuromuscular control. Overall, a comprehensive approach that combines strength, kinematics, balance, and psychological readiness offers the most effective framework for monitoring recovery, predicting injury risk, and optimizing safe return to sport.

Despite its strengths, this review has several limitations. First, the included studies varied in methodology, sample characteristics, and biomechanical assessment tools, which may limit the comparability and generalizability of the findings. Second, most of the research was cross-sectional or retrospective, precluding causal inferences regarding injury risk or recovery. Additionally, some studies lacked control for potential confounders such as training load, prior injury history, or exposure time, which could affect outcome interpretation. Finally, publication bias may be present, as only English-language, peer-reviewed journal articles were included.

## Conclusions

The evidence indicates that biomechanical screening tools - particularly those assessing muscle strength symmetry, landing kinematics, and postural control - provide valuable insights into injury risk and readiness for return to sport. Psychological readiness further complements these assessments, underscoring the multifactorial nature of rehabilitation. However, caution is warranted when relying on proprietary metrics or single tests in isolation. Future research should prioritize longitudinal and multicenter studies using standardized protocols to develop reliable, predictive models for injury risk and recovery outcomes in athletes.
